# The Intracerebral Haemorrhage in Patients With Dengue Fever: A Systematic Review and Meta‐Analysis

**DOI:** 10.1002/rmv.70060

**Published:** 2025-08-07

**Authors:** Mingxia Xu, Ming Dong

**Affiliations:** ^1^ Otolaryngology & Head and Neck Center Cancer Center Department of Head and Neck Surgery Zhejiang Provincial People's Hospital Affiliated People's Hospital Hangzhou Medical College Hangzhou China; ^2^ Otolaryngology & Head and Neck Center Cancer Center Department of Nursing, Zhejiang Provincial People's Hospital Affiliated People's Hospital, Hangzhou Medical College Hangzhou China; ^3^ Department of Intensive Care Unit Huzhou Nanxun People's Hospital Huzhou Zhejiang China

**Keywords:** dengue, intracerebral haemorrhage, meta‐analysis, systematic review

## Abstract

Dengue virus is a neurotropic virus capable of infecting the supporting cells of the central nervous system. One of the most severe neurological consequences of this infection is intracerebral haemorrhage, which is a leading cause of death worldwide. This study aimed to systematically review and analyse the existing literature on this topic, providing insights into the potential neurological consequences for patients with dengue fever. A comprehensive search was conducted across the PubMed, Scopus, Web of Science, and Embase databases to extract relevant published data up to February 2025. This meta‐analysis included articles that were designed as cohort studies. A critical appraisal was conducted using the Newcastle–Ottawa Scale (NOS) score. Risk was employed as a measure of pooled effect size based on a random‐effects model. Heterogeneity was assessed using the Q test and the I^2^ index. This meta‐analysis included 6 studies involving a total of 2861 individuals who directly assessed the risk of intracerebral haemorrhage. The reported risk of intracerebral haemorrhage was 14 cases per 1000 dengue fever patients [0.014 (95% CI: 0.002, 0.026), *p* = 0.020, I^2^ = 94.64%]. Notably, prospective studies with low methodological quality indicate a higher risk of intracerebral haemorrhage compared to retrospective studies and those of high quality. Given the high risk of intracerebral haemorrhage in patients with dengue fever, it is essential for physicians to evaluate affected individuals for the potential occurrence of cerebral haemorrhage.

## 1 Introduction

Dengue virus infection poses a significant health threat, affecting at least 3.6 billion people across more than 125 countries in tropical and subtropical regions who are at high risk [1, 2]. Dengue is the most rapidly spreading mosquito‐borne disease in worldwide and recent study estimated the global burden of dengue to be approximately 390 million infections per annually [3]. Dengue is a febrile illness with clinical manifestations ranging from asymptomatic infection to severe infection with multi‐organ dysfunction. A majority of patients with dengue fever (DF) recover without complication, but the mortality rate ranges from 1% to 5% without treatment, but it decreases to less than 1% when adequate treatment is administered [4, 5, 6].

However, dengue haemorrhagic fever (DHF), also known as severe dengue, presents a wide spectrum of bleeding manifestations and carries a mortality rate of 26% [7]. Neurological involvement, which may include encephalopathy, intracerebral haemorrhage (ICH), or infarction, is a rare but potentially fatal complication of dengue [8, 9]. The incidence of ICH in dengue infections is approximately 1.1% [10].

ICH, a condition characterised by bleeding within the brain, is a leading cause of morbidity and mortality worldwide [11]. Dengue virus is a neurotropic virus with the ability to infect the supporting cells of the central nervous system (CNS). Neural injury during the acute stage of the infection results from direct neuro‐invasion and/or the phenomenon of antibody‐dependent enhancement, resulting in plasma leakage and coagulopathy [12].

Dengue fever poses a significant public health challenge due to the potentially fatal outcomes associated with severe infections, including ICH. Consequently, assessing the risk of ICH and implementing appropriate clinical management strategies are crucial for preventing mortality related to dengue fever. The objective of this article is to systematically review and analyse the existing literature on this subject, offer insights into the risk of potential neurological complications of dengue, particularly ICH, and identify gaps in research that could inform clinical practice and public health strategies.

## 2 Method

The PRISMA statement was followed in conducting this meta‐analysis [13]. We performed a comprehensive literature search using the online databases PubMed, Scopus, Web of Science, and Embase, covering publications up to April 2025. The search terms included ‘Intracerebral Haemorrhage OR Cerebral Haemorrhage OR Cerebrum Haemorrhage OR Cerebral Brain Haemorrhage OR Cerebral Parenchymal Haemorrhage’ and ‘Dengue Fever OR Classical Dengue OR Classical Dengue Fever OR Break Bone Fever’ which the used to identify literature.

### 2.1 Inclusion and Exclusion Criteria

Studies were included for further evaluation if they met the following criteria [1]: publications in English [2]; investigation of ICH, including subarachnoid haemorrhage, intraparenchymal haemorrhage (IPH), subdural haemorrhage (SDH), or haemorrhage related to strokes and venous thrombosis [3]; determination of the association between ICH and dengue fever; and [4] evaluation of risk or incidence, along with their corresponding 95% confidence intervals [14], or sufficient data was provided to assess these associations. Studies were excluded based on the following criteria: non‐English articles; other types of publications such as conference proceedings, abstracts, reviews, or meta‐analyses; and insufficient data to calculate risk and 95% CIs.

### 2.2 Data Extraction and Quality Evaluation

All included studies were meticulously identified by two investigators, and any uncertain data were reviewed by a third author. The following information was collected: the first author's name, publication year, nationality, sample type, assay used to evaluate the expression of ICH, sample size, type of ICH, and the associated risk along with their 95% confidence intervals [14].

### 2.3 Quality Appraisal

The quality assessment of the included studies was conducted using the Newcastle‐Ottawa Scale (NOS). The NOS employs a 'star system' to evaluate three criteria: selection, comparability, and exposure. Studies could receive a maximum of four stars for selection, three stars for exposure, and two stars for comparability. In total, the methodological quality of each study was rated on a scale from 0 to 9 stars, with a higher star count indicating superior methodological quality. Furthermore, the quality assessment was carried out by two independent researchers, and any disagreements were resolved by a third party [15].

### 2.4 Statistical Analysis

The association between dengue fever and ICH was evaluated using Stata software version 17, utilising a random effects model (StataCorp, 2024, Stata Statistical Software: Release 17, College Station, TX). The risk of ICH and the corresponding 95% confidence intervals [14] were collected to evaluate the risk of ICH in individuals infected with dengue fever.

Heterogeneity was assessed using the Q test and the I^2^ index. Studies with an I^2^ index of less than 25%, between 25% and 75%, and greater than 75% were categorised as having low, moderate, and high heterogeneity, respectively. In cases where the I^2^ index exceeded 75%, a subgroup and meta‐regression analysis was conducted based on the types of study design and level of quality. Forest plots were utilised to visualise the risk in each study, along with the estimated values and their corresponding 95% confidence intervals (95% CI) in both the main analysis and the subgroup analysis. Publication bias of the meta‐analysis was determined by Begg's test and Egger's test.

## 3 Result

### 3.1 Search Results

**FIGURE 1:**
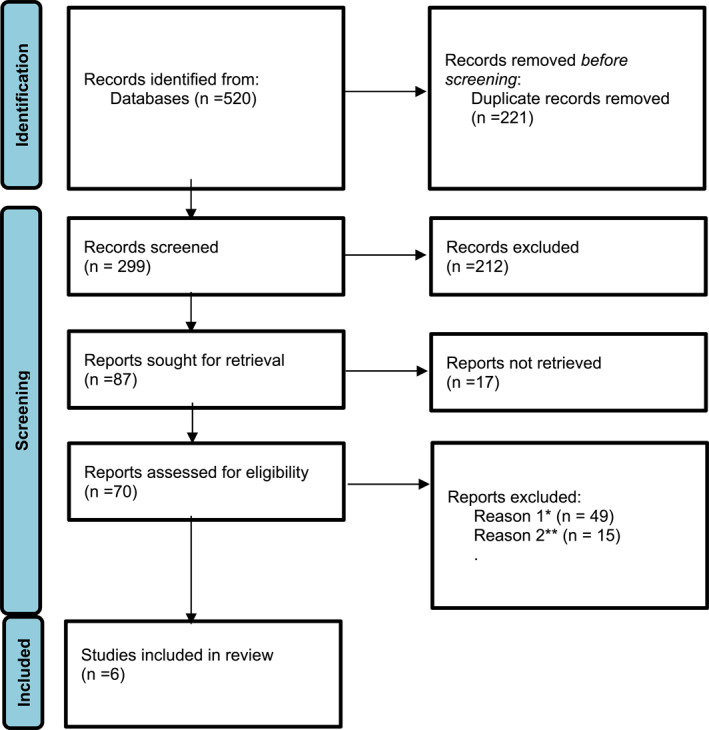
PRISMA flowchart of study selection process. *Reason 1 = case reports, case series, and review articles. **Reason 2 = case‐control and RCT article.

A total of 520 articles were retrieved from the databases PubMed, Scopus, Web of Science, and Embase during the initial search. We identified 192 papers from Medline/PubMed, 143 papers from Scopus, 133 papers from Web of Science, and 52 papers from Embase. After removing duplicates, case reports, case series, review articles, and meta‐analyses, as well as studies that were not written in English or were unrelated, 70 records were deemed eligible for full‐text review. Only 6 papers reported the risk of ICH in patients with dengue fever. These 6 papers underwent quality assessment and were included in our meta‐analysis (Figure 1). The overall sample size comprised 2861 patients, with individual studies ranging from 92 to 1148 participants.

### 3.2 Main Characteristics

The six selected articles, which involve a sample size of 2861 participants, have been published across four continents: Taiwan, Pakistan, India, and Nepal. The individuals involved in the studies were people infected with dengue fever who were referred to hospitals or other medical and research centres for treatment. Necessary tests were conducted to diagnose any brain and neurological damage. Table 1 presents a comprehensive overview of the key characteristics of the included studies. All articles were designed as cohort studies, incorporating both retrospective and prospective approaches. The lowest and highest risks of ICH were 1 and 45 cases per 1000 dengue fever patients, respectively.

**TABLE 1:** Baseline characteristics of the included studies in the systematic review and meta‐analysis.

Authors	Year	Country	Study design	Sample size	No. ICH	Risk of ICH	95% CI^a^		Overall quality assessment (NOS Score^b^)
							Lower	Upper	
Chang, et al. [16]	2021	Taiwan	Retrospective cohort	182	3	0.016	−0.002	0.035	6
Assir, et al. [17]	2020	Pakistan	Retrospective cohort	92	3	0.033	−0.004	0.069	7
Kulkarni, et al. [18]	2021	India	Retrospective cohort	154	7	0.045	0.013	0.078	7
Mathew, et al. [19]	2022	India	Retrospective cohort	1145	3	0.003	0.000	0.006	8
Sahu, et al. [20]	2020	India and Nepal	Prospective cohort	486	11	0.023	0.009	0.036	8
Koshy, et al. [21]	2021	India	Prospective cohort	799	1	0.001	−0.001	0.004	8

^a^ Confidence Interval.

^b^ Newcastle–Ottawa Scale.

### 3.3 Meta‐Analysis

**FIGURE 2:**
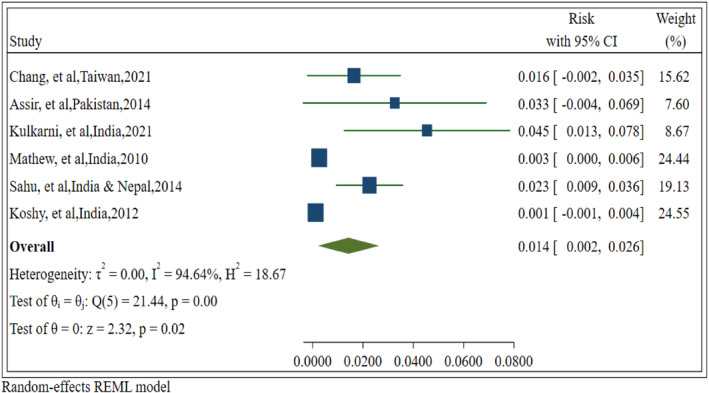
Forest plot of the pooled risk of intracerebral haemorrhage in dengue fever patients. CI, confidence interval.

A random‐effects model was employed to calculate the pooled risk ICH. The overall risk of ICH was determined to be 14 cases per 1000 dengue fever patients [0.014 (95% CI: 0.002, 0.026), *p* = 0.020]. Significant heterogeneity was observed among the included studies (Q‐value: 18.67, df = 5, z‐value = 2.32, I^2^ = 94.64%, *p*‐value ˂ 0.001). However, a subgroup analysis was conducted to explore the research in greater detail based on the methodological quality and study design (Figure 2).

### 3.4 Subgroup Analysis

**FIGURE 3:**
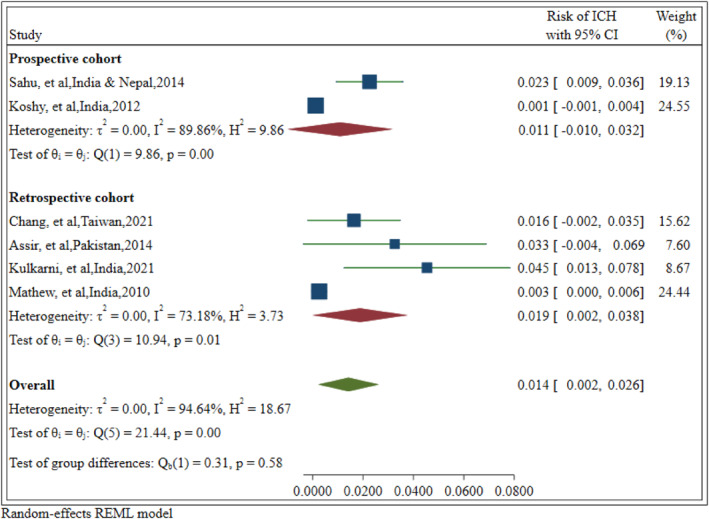
Forest plot illustrating the pooled risk of intracerebral haemorrhage in patients with dengue fever, categorised by study design. CI, Confidence Interval.

**FIGURE 4:**
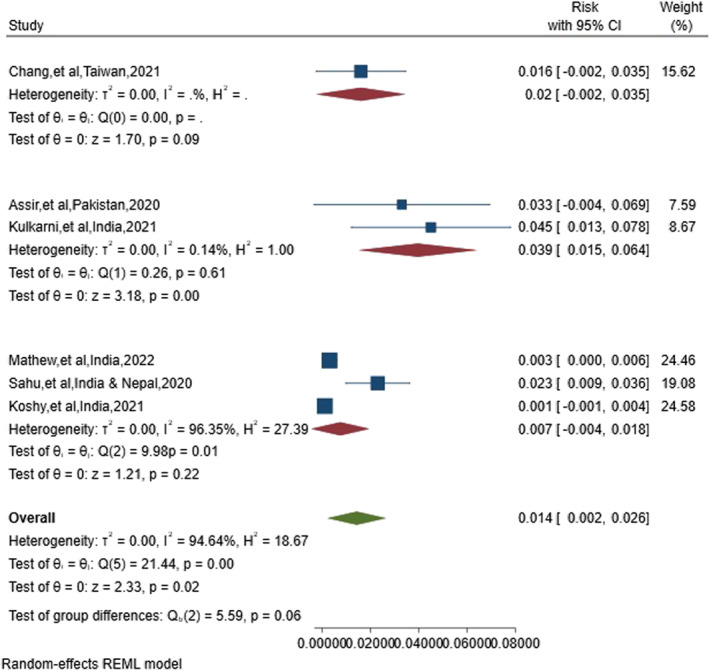
Forest plot illustrating the pooled risk of intracerebral haemorrhage in patients with dengue fever, categorised by methodological quality. CI, Confidence Interval.

Subgroup analyses were conducted based on the methodological quality (6, 7, and 8 stars) and study design (retrospective and prospective cohort) for further investigation. Regarding the significant subgroups of the included studies, lower‐quality articles reported a higher risk of ICH. Studies that collected data retrospectively found a greater incidence of ICH in patients with dengue fever (19 cases vs. 11 cases per 1000 patients). The findings of the subgroup analysis are presented in Table 2 and Figures 3 and 4.

**TABLE 2:** Subgroup analysis assessing the role of various variables in the risk of intracerebral haemorrhage among patients with dengue fever.

Subgroup	Number of studies	Risk of ICH (95% CI^a^)	*p*‐value	Test of heterogeneity	
				I^2^	*p*‐value
Overall results	6	0.014 (0.002, 0.026)	0.020	94.64	< 0.001
Level of quality (number of stars)					
6	1	0.016 (−0.002, 0.035)	0.082	—	—
7	2	0.039 (0.015, 0.064)	0.001	0.14	0.608
8	3	0.007 (−0.004, 0.018)	0.228	96.35	0.007
Study design					
Prospective cohort	2	0.011 (−0.010, 0.032)	0.307	89.86	0.002
Retrospective cohort	4	0.019 (0.002, 0.038)	0.050	73.18	0.012

^a^ Confidence Interval.

### 3.5 Meta‐Regression

Meta‐regression analysis revealed a statistically significant association between the risk of ICH with methodological quality and study design. Specifically, for each one‐level increase in the quality of the article, the risk of reporting ICH decreased by 8 cases per 1000 individuals [−0.008 (95% CI: −0.032, 0.016), *p* = 0.508], as well as prospective studies indicate a higher risk of ICH in 8 out of 10,000 individuals with dengue fever when compared to retrospective studies [0.0005 (95% CI: −0.037, 0.038), *p* = 0.976]. Following the meta‐regression analysis that included significant variables, the heterogeneity among the studies was generally reduced (Q‐value: 5.73, df = 3, I^2^ = 82.53%, *p*‐value = 0.001). Detailed results of the meta‐regression are presented in Table 3.

**TABLE 3:** A random meta‐regression analysis of covariates associated with the risk of intracerebral haemorrhage in patients infected with dengue fever.

Moderators	β‐ coefficient	SE^a^	Z value	95% CI^b^	2‐ Sided *p* value
Level of quality (number of stars)	−0.008	0.012	−0.66	−0.032 to 0.016	0.508
Study design	0.0005	0.019	0.03	−0.037 to 0.038	0.976

^a^ Standard error.

^b^ Confidence Interval.

### 3.6 Publication Bias

Publication bias was not detected in the meta‐analysis using Begg's test (*p* = 0.259), but significant publication bias was observed in the Egger's test (*p* < 0.001). Significant heterogeneity was observed in the meta‐analysis (Q_(5)_ = 18.67, *p* < 0.001).

## 4 Discussion

A total of 2861 patients with dengue fever patients were included in this systematic review and meta‐analysis, which comprised 6 articles that directly assessed the risk of ICH. All included studies employed a cohort design. The results indicated that the risk of ICH in patients with dengue fever patients was elevated, and this risk was found to be statistically significant. The methodological quality and the study design were identified as variables affecting the risk of ICH.

ICH in patients with dengue has rarely been reported. Reported on 799 patients with dengue fever, 21 of whom (0.5%) presented with central nervous system involvement, but only one of which had ICH [21]. Reported 9 cases of intracranial bleeding in patients with dengue, however, no incidence rate was reported [22]. The reported risk of ICH in this study was 14 cases per 1000 dengue patients, with a range of 1–45 cases. There was variability in the risk of ICH among individuals with dengue fever across the included studies. This variability may reflect differences in the patient populations studied; the highest risks were reported in cohorts of critically ill patients and in studies examining the neurological complications of dengue fever.

Most studies on ICH in patients with dengue fever are limited to examining individuals with ICH and are primarily conducted as case reports or case series. The objective of these studies was not to estimate the risk or incidence of ICH in this patient population [10, 16]. However, the studies included in this systematic review and meta‐analysis reported the number of individuals with dengue fever who developed ICH. Two prospective [20, 21] and four retrospective [16, 17, 18, 19] studies were reviewed. The results of the meta‐regression model indicated that prospective studies, which monitor patients more closely for research purposes and are less likely to record events with bias, reported a higher risk of ICH.

The results presented in Table 2 indicate that lower‐quality studies and retrospective studies reported a higher risk of intracranial haemorrhage (ICH) in individuals with dengue fever. However, in the meta‐regression model shown in Table 3, where both study quality and study design were included simultaneously, studies with prospective designs reported a higher risk of ICH. This discrepancy may be attributed to the potential for measurement, confounding, and selection biases, which are more prevalent in retrospective studies [23, 24, 25].

The current study underscores the risk of ICH on dengue patients and the limited information available to medical practitioners. Timely diagnosis and intervention can save lives. Clinicians should remain vigilant for the possibility of ICH in dengue patients, particularly when they present with altered consciousness. Dengue infection in 2014 was identified as an independent risk factor for ICH, suggesting that viral pathogenesis may also contribute to the development of intracranial haemorrhage in these patients. The threshold for recommending a diagnostic brain CT scan should be lowered for dengue patients exhibiting altered consciousness, especially during years when a higher frequency of infarction and ICH has been observed [16].

## 5 Conclusion

This systematic review and meta‐analysis confirms that patients with dengue fever are at an increased risk of developing intracranial haemorrhage (ICH), a finding that is statistically significant. Although the incidence of ICH among dengue patients has historically been low, the results underscore the importance of recognising the potential for ICH, particularly in critically ill patients or those presenting with altered levels of consciousness. The study further emphasises that prospective studies, which are less susceptible to bias, reported a higher risk of ICH than retrospective studies. This underscores the importance of careful monitoring and timely diagnosis. In light of these findings, clinicians should remain vigilant, particularly in cases where dengue patients exhibit neurological symptoms. A lower threshold for recommending a brain CT scan may be warranted, especially during periods of heightened risk for complications such as ICH and infarction. Additionally, the findings suggest that viral pathogenesis may contribute to the development of ICH, further supporting the need for ongoing research into the underlying mechanisms of dengue‐related neurological complications.

## Author Contributions

**Mingxia Xu:** writing – original draft and analyses the data. **Ming Dong:** conceptualisation, review and editing, data curation, validation.

## Conflicts of Interest

The authors declare no conflicts of interest.
